# Enhancing radiator cooling capacity: A comparative study of nanofluids and water/EG mixtures

**DOI:** 10.1016/j.heliyon.2024.e38352

**Published:** 2024-09-26

**Authors:** Tugba Tetik, Yasin Karagoz

**Affiliations:** Istanbul Medeniyet University, Department of Mechanical Engineering, Istanbul, Turkiye

**Keywords:** Trihybrid nanofluids, Heat transfer, Radiator, Thermal imaging

## Abstract

The present study experimentally investigates and compares the performance of a radiator system cooled by a water-ethylene glycol (70:30) based Al_2_O_3_-SiO_2_-TiO_2_ nanofluid, with that of a radiator cooled by a water/EG mixture. The equal pumping power criterion of the pump with an equal mass flow rate was used for comparison. Mass flow rate and nanoparticle volume fraction on a radiator cooling system and the radiator's capacity have been studied. Five different nanofluids were prepared with different composition ratios of Al_2_O_3_-SiO_2_-TiO_2_ with a total of 0.45 % nanoparticles. The flowrate changed in the range of 0.02–0.032 kg/s. The results showed that the increase in heat transfer is mainly due to the flow velocity and the nanoparticles added in different proportions to the base liquid. The UA value and enhancement ratio of NF1 compared to EG/W is 14–18.5 %, for NF2 it is 14.9–21.8 %, for NF3 it is 15.1–23.4 %, for NF4 it is 15.6–27.5 %, and for NF5 it is 15.9–30 % at 0.02 kg/s and 0.032 kg/s. According to the experimental study results, nanofluids with low concentrations of nanoparticles can enhance the heat transfer rate up to 30 % as a comparison with water/EG.

## 1 Introduction

Nanofluids, which are fluids containing nanoparticles, typically less than 100 nm in size, increase the thermal conductivity of the coolant [[1], [2], [3], [4], [5]]. This increase is also associated with heat transfer [6,7]. Numerous studies have shown that nanofluids improve heat transfer properties in a variety of applications including automobile radiators. The efficiency of the radiator's cooling function relies on the coolant, which absorbs energy from the engine. Nanofluids have shown promising results in increasing heat transfer in radiators, where optimum cooling is crucial for engine performance. Improved heat transfer capability using nanofluids allows the radiator to remove heat from the engine more effectively [[8], [9], [10]]. One of the methods used to measure the effectiveness of the coolant used in the radiator is to examine the temperature distribution of the radiator by using a thermal camera [[11], [12], [13]].

In nanofluid studies on automobile radiator cooling systems, water is generally used as the base fluid, but the use of water and ethylene glycol mixtures is also frequently encountered [7]. The proportion of ethylene glycol and water in a coolant is determined based on factors such as the desired freeze protection, boil-over protection, and overall thermal performance of the coolant. In moderate climates, it is generally recommended to use a coolant mixture with a lower concentration of ethylene glycol, such as around 30 %–40 % ethylene glycol and 70 %–60 % water. When determining the nanoparticles to be used for specific heat transfer applications, factors such as operating conditions, desired improvements and cost considerations are considered. As nanoparticles, there are heat transfer studies made with different types such as metallic nanoparticles, carbon nanotubes, and metal oxide nanoparticles [9,[14], [15], [16], [17]]. The concentration of nanoparticles ranges from very dilute concentrations to highly concentrated suspensions. Thermal testing of nanofluids encompasses various methods to evaluate their heat transfer properties and compare them with conventional fluids. One such method is heat exchanger testing, which is of great importance for practical applications, as it allows for the assessment of how nanofluids perform in real-world systems. The literature includes both experimental and numerical studies that examine the radiator performance of hybrid and ternary nanofluids [18,19].

Zubair et al. investigated the heat transfer of an engine radiator using TiO_2_/EG-water nanocoolant [20]. The study experimentally determined the effectiveness of using a nanofluid composed of water and ethylene glycol as a radiator coolant. Using nanocoolant with a nanoparticle concentration of 0.03 % resulted in a 29.5 % increase in heat transfer compared to water at a flow rate of 150 LPH. However, no further heat transfer enhancement was observed with varying nanoparticle concentrations in the base fluid. Nieh et al. studied the use of TiO_2_ and Al_2_O_3_-based nanocoolants in an air-cooled radiator to improve its performance [21]. Nanocoolants were prepared using a two-step method with varying concentrations (0.5 wt%, 1.0 wt%, and 2.0 wt%). The findings revealed that the TiO_2_-based nanocoolant had a higher heat removal capacity than the Al_2_O_3_-based nanocoolant. The maximum improvement in heat dissipation capacity was 25.6 %. Hussein et al. concluded that in their study with TiO_2_ and SiO_2_ nanoparticles, SiO_2_, which has a lower density, increases heat transfer more than TiO_2_ due to its higher average speed [16]. Marulasiddeshi et al. studied the synthesis, characterization of the Al_2_O_3_ and CuO nanoparticles and entropy generation and exergy analysis of hybrid nanofluids in a tube subjected to CHF conditions [22,23]. The study observed that water-based Al_2_O_3_ nanofluids enhance thermal conductivity by 8.6 % for 1 vol% at 60 °C. Ramadhan et al. studied Al_2_O_3_-TiO_2_-SiO_2_ nanofluid in the ratio of 0.05, 0.1 %, 0.2 %, and 0.3 % to a mixture of water and ethylene glycol. In their tests, they concluded that there was a significant improvement in thermal conductivity with increasing nanoparticle ratio [24]. Ramadhan et al. investigated the thermal conductivity and stability of tri-hybrid nanofluid. With the addition of tri-hybrid nanoparticles, stability was achieved and a significant improvement in heat transfer was achieved [25]. Ramadhan et al. investigated the cooling performance of tir-hybrid nanoparticles in automotive radiators and concluded that the cooling performance improved significantly with increasing amount of nanoparticles [26]. Fikri et al. examined the effect of mixing TiO2-SiO2 nanofluid in different ratios between 0.3 % and 1 % on stability and they were able to provide stability for up to 10 days [27]. Ramadhan et al. investigated the effect of Al2O3-TiO2-SiO2 nanoparticle addition on the automotive radiator cooling system and a significant improvement in heat transfer was observed [28].

Also, ultrasonication improves the stability and heat transfer properties of nanofluids [29]. Ramadhan et al. studied the stability of tri-hybrid nanofluid containing Al_2_O_3_-SiO_2_-TiO_2_ suspended in a Water-Ethylene Glycol (EG) mixture using UV–Vis, zeta potential, sedimentation, and micrograph observation techniques [30]. In order to obtain a stable suspension, researchers suggested that the optimal time for sonication is 10 h. Authors examined the impact of nanoparticle composition ratio on dynamic viscosity in a separate study [31]. Kanti et al. studied the stability of SiO_2_ and TiO_2_ nanoparticles with graphene oxide when dispersed in distilled water. They reported excellent stability (30 days), with surfactant PVP (polyvinylpyrrolidone) [32].

<comment>Based on the literature review, it has been concluded that</comment><comment> </comment>hybrid nanofluids containing Al_2_O_3_-SiO_2_-TiO_2_ offer good stability. Furthermore, while there are studies in the literature that use Al_2_O_3_, SiO_2_, and TiO_2_ separately, the purpose of this study is to investigate their combined cooling performance across a wide range of ratios. In this study, nanoparticles were tested in a stable suspension state at ratios up to 0.45 % in a 70:30 water:EG condition, which differs from previous literature studies. The study also examined the ratios of Al_2_O_3_, SiO_2_, and TiO_2_ that make up the trihybrid fluid in a wider range than previous literature. Our study will experimentally determine heat transfer properties and evaluate their suitability as coolants in radiators. The current study estimates the cooling performance of a radiator in which conventional coolant is replaced by nanofluids. The study focuses on the effects of the mass flow rate and nanoparticle volume fraction on the radiator cooling system and the radiator's capacity. For the preparation of nanofluids, Al_2_O_3_-SiO_2_-TiO_2_ nanoparticles were dispersed in water:EG solution. Heat transfer for the radiator was modelled using LMTD method and results were compared with the base fluid. Also, temperature distribution was examined with a thermal camera.

## 2 Methodology

### 2.1 Materials

Tri-hybrid nanofluids were prepared at 0.45 % weight concentrations for various composition ratios of nanoparticles. Three types of nanoparticles (Al_2_O_3_-SiO_2_-TiO_2_) were selected. A 70:30 ratio of water: ethylene glycol was used to synthesize solutions. Non-ionic surfactant PVP (polyvinylpyrrolidone) used to ensure stability. The same amount of surfactant were used in all coolants to prevent any potential impact on the properties of the nanofluid.

Table 1 lists the specifications provided by the supplier of each of the nanofluid components below.

**Table 1:** Properties of the nanofluid components [33,34].

Properties	Al_2_O_3_	SiO_2_	TiO_2_	Ethylene Glycol
**Purity**	99.5+%	99.5 %	99.5+%	99.5 %
**Density [kg/m**^**3**^**]**	3900	2400	4100	1100
**Average Particle Size (nm)**	18	10–20	200	–

### 2.2 Preparation of nanofluids

In the preparation of nanofluids, the initial step involves determining the required quantity of nanofluid based on the system components. The nanoparticles for nanofluid preparation using the two-step method were identified, and their requisite amounts were calculated accordingly. In this process, nanoparticles are first obtained through either purchase or production using various methods. These dry nanoparticles are then dispersed into the base fluid via techniques such as magnetic stirring or ultrasonication [35]. The solutions are subsequently stirred and placed in an ultrasonic bath. Stability is assessed through sedimentation analysis.

For the preparation of the coolant to be used in the radiator system, three types of metal oxides were selected based on their thermal conductivity: high thermal conductivity (Al_2_O_3_), medium thermal conductivity (TiO_2_), and low thermal conductivity (SiO_2_).

Nanofluids containing Al_2_O_3_-SiO_2_-TiO_2_ were prepared using a 70:30 water: ethylene glycol ratio. PVP was selected as the surfactant for the test liquids due to its superior stability than SDS [36]. All components were weighed on a high-precision balance with a max capacity of 220g (Shimadzu ATX224). Nanofluids with 0.45 % nanoparticle volume fraction were formulated by mixing and vibrating the nanoparticles, surfactant (in a 1:10 ratio of nanoparticles [37]) and base fluid.

The percentage ratios of nanoparticles, which are 0.45 % by volume in the nanofluid, are given in Table 2 for each sample.

**Table 2:** Composition ratio of nanoparticles.

Sample No	Nanoparticle Proportions (%)		
	Al_2_O_3_	SiO_2_	TiO_2_
NF1	10	60	30
NF2	30	60	10
NF3	33.33	33.33	33.33
NF4	60	30	10
NF5	60	10	30

### 2.3 Nanofluid properties

The density of nanofluids depending on the varying volume fractions were estimated by mixture model.

$$\rho =\frac{m_{np}+m_{bf}}{V_{np}+V_{bf}}=\varphi \rho_{np}+\left(1-\varphi \right)\rho_{bf}$$

In the equation m_np_ is the mass of nanoparticles which is the sum of Al_2_O_3_-SiO_2_-TiO_2_ and $m_{bf}$ is the mass of base fluid ethylene glycol and water. φ is the volume fraction.

$${m_{np}=m}_{Al2O3}+m_{TiO2}+m_{SiO2}$$

$$m_{bf}=m_{EG}+m_{water}$$

The heat capacity (c) is calculated assuming thermal equilibrium between the nanoparticles and the base fluid [38].

$$c_{\;nf}=\frac{\varphi \rho_{np}c_{np}+\left(1-\varphi \right)\rho_{bf}c_{bf}}{\rho_{nf}}$$

### 2.4 Physical characterization and stability control

One of the most important properties of nanofluids for reliable use in industrial applications is stability. Nanofluids with good stability and dispersibility will reduce the risk of sedimentation and clogging in the cooling system.

**Fig. 1:**
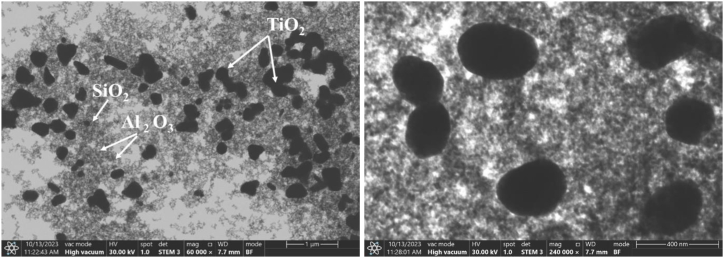
SEM image of the nanofluid at 60000X and 240000X.

In the experimental study, surfactant adding and ultrasonication techniques were used to ensure stability. Following the sample preparation, the microstructure and morphology of the hybrid nanofluids was investigated with electron microscopy techniques. The scanning electron microscopy image of the nanofluid acquired by mixing three nanoparticles - Al_2_O_3_, SiO_2_, and TiO_2_ - demonstrates structural features. The SEM images of trihybrid nanofluids dispersed in a water:EG mixture are presented in Fig. 1, which displays the distribution and sizes of all three nanoparticles. In the images given for 60000X and 240000× magnification ratios, the acceleration voltage is set to 30 kV.

The nanoparticle composition highlights the spherical morphology of Al_2_O_3_, nearly spherical particle shape of SiO_2_, and elongated structure of TiO_2_. Furthermore, the image demonstrates the nanoparticles' uniform dispersion throughout the nanofluid, indicating excellent stability and potential for various applications.

**Fig. 2:**
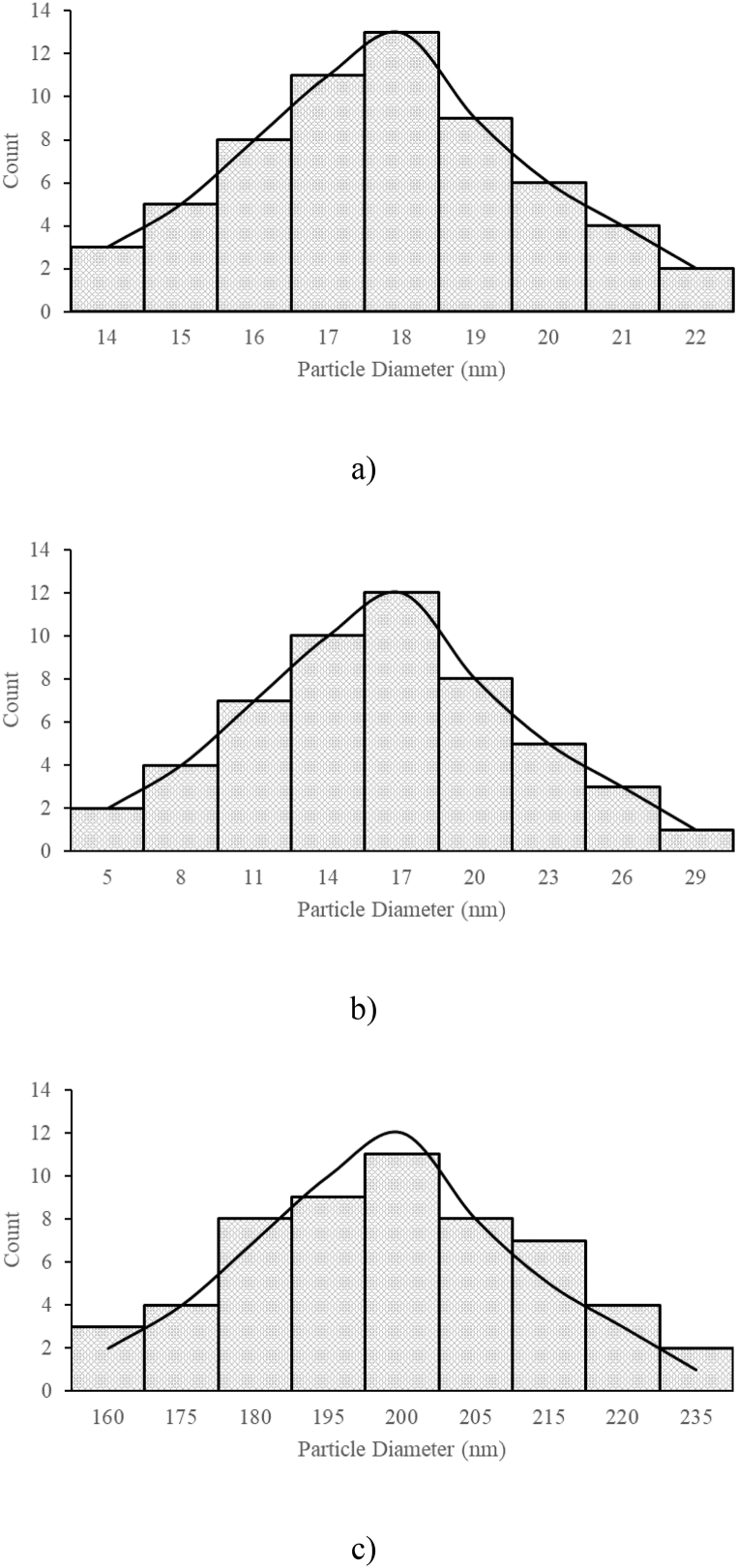
Particle size distributions of a) Al_2_O_3_, b) SiO_2_ and c)TiO_2_.

Size distributions were also plotted for the nanoparticles used. Fig. 2 demonstrates that Al_2_O_3_, SiO_2_ and TiO_2_ nanoparticle sizes vary between 14 and 22 nm, 5–29 nm, 160–235 nm, respectively.

**Fig. 3:**
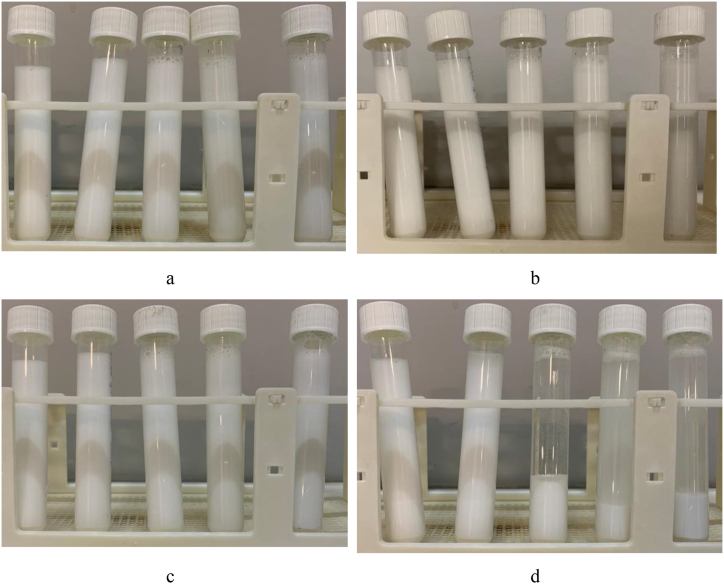
Sedimentation tests for samples. a) t = 0 b) t = 60 min c) t = 48 h and d) 72 h.

Long term stability analysis was conducted to assess how the nanofluid maintains its properties over time. Visual inspection is a fundamental method for assessing the stability of nanofluids, focusing on the observable characteristics of the fluid over time and is used to detect any visible signs of instability, such as sedimentation or particle aggregation. To perform sedimentation tests, each sample was placed in test tubes of the same diameter and observed over time. Fig. 3 shows sedimentation tests of nanofluid samples for 72 h. Based on the figure, NF1 has the longest stability among the samples.

Blending nanoparticles with water:EG solution resulted in an increased surface area and collisions, consequently improving the heat transfer capabilities of the nanofluid [39].

### 2.5 Heat transfer analysis

The total heat transfer in the radiator was determined by using the overall heat transfer coefficient (U) and the temperature differences between the fluids (air and coolant).

Assuming that the heat exchange is only between the coolant and the air, the energy balance can be obtained using the first law of thermodynamics as follows:

$$\dot{Q}={\dot{m}}_{air}c_{p,air}{\Delta T}_{air}={\dot{m}}_{coolant}c_{coolant}{\Delta T}_{coolant}$$

By measuring the inlet and outlet temperatures of the two fluids in the system, it is possible to apply the Log Mean Temperature Difference (LMTD) method for the heat exchanger [40].

$$\dot{Q}=UA{\Delta T}_{lm}$$

Logarithmic mean temperature difference;

$${\Delta T}_{lm}=\frac{{\Delta T}_{1}-{\Delta T}_{2}}{\ln\left(\frac{{\Delta T}_{1}}{{\Delta T}_{2}}\right)}$$

For a cross-flow heat exchanger temperature difference is corrected with a factor, F

$$F=\frac{\ln\left(\frac{1-RS}{1-S}\right)}{\left(1-\frac{1}{R}\right)\ln\left(1+Rln\left(1-S\right)\right)}$$

R and S parameters in the equation are;

$$R=\frac{T_{a,o}-T_{a,i}}{T_{c,i}-T_{c,o}}$$

$$S=\frac{T_{c,o}-T_{c,i}}{T_{a,i}-T_{a,o}}$$

In this study, cooling performance is defined with UA (global coefficient of heat transfer).

$$UA=\frac{{\dot{m}}_{c}c_{p,c}\left(T_{c,i}-T_{c,o}\right)}{F{\Delta T}_{lm}}$$

The UA value was utilised to assess the effectiveness of the cooling method with various nanocoolants in terms of heat transfer. This method has been employed in analogous research studies within the literature [41,42].

### 2.6 Experimental setup

Heat exchanger method for thermal testing of nanofluids involves filling a heat exchanger with nanofluid and measuring its performance in terms of heat transfer efficiency. Before introducing the nanofluid, baseline tests are conducted using a conventional base fluid to establish reference performance metrics. During testing, parameters such as flow rates and inlet temperatures are varied to assess their impact. Analyzing the results provides a comparative analysis to illustrate the benefits of using nanofluids in heat exchangers and helps understand the practical viability of nanofluids in enhancing heat exchanger performance.

This study presents an experimental study of cases in which the nanofluids given in Table 2 are cooled with air in a radiator. UA was calculated under known conditions of the inlet and outlet properties of both fluids.

**Fig. 4:**
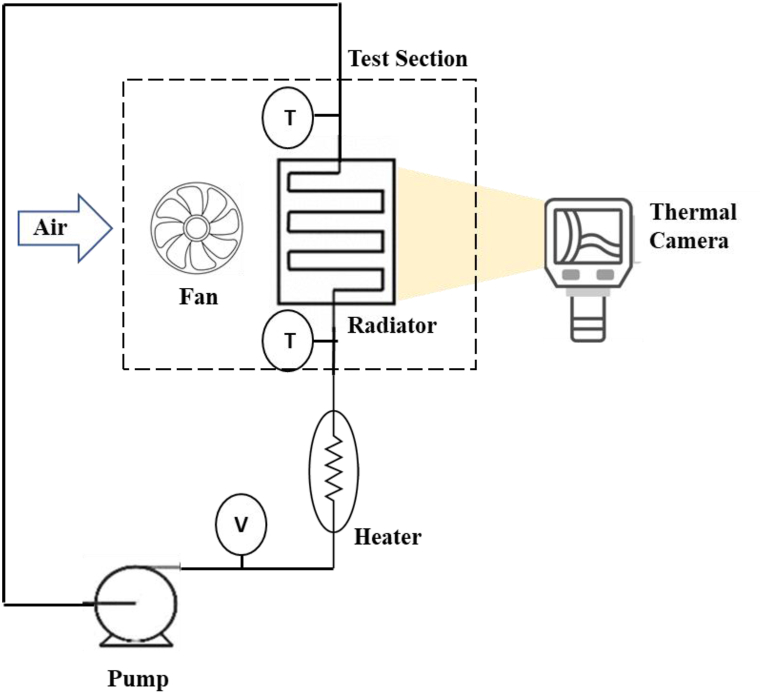
Experimental set-up.

Fig. 4 illustrates the schematic for the study. A total volume of 260 mL was formed with a volume concentration of 0.45 % for tri-hybrid nanofluids. Prepared nanofluids were used as coolant fluid. Heat transfer experiments were performed using nanofluids prepared in the ratios specified in Section 1.2. The effect of flow rate and nanoparticle concentration were examined and results were compared to base liquid.

Temperature measurements for coolant were taken with the pump functioning at three distinct flow rates in the range of 0.02–0.032. Two cartridge heaters with a total output of 96 W were used to simulate the thermal energy released by combustion in the engine. The radiator's active area is a square with sides measuring 120 mm. A 120 mm wide fan with a constant air speed of 4.3 m/s was used for heat dissipation in the heat exchanger.

Temperatures were measured at five different points, including the temperatures of the air and coolant inlets and outlets, as well as ambient temperature. In addition, images of the radiator and fan were recorded with a thermal camera during each experiment. The thermal heat analysis was carried out by recording the images of radiator using thermal camera Fluke TiS60 model. The heat distribution of the radiator and the coolant in the radiator were analyzed from the captured images.

### 2.7 Test procedure

The study examines the thermal performance of a radiator employing ethylene glycol-water based coolant. The procedure used for the study is described below.

• The effect of varying proportions of nanoparticles on the performance of the radiator.

The total heat transfer in the radiator was calculated by maintaining constant flow rates of coolant and air.

• The effect of coolant flow rate on the performance of the radiator.

The radiator's total heat transfer was calculated for two different coolant flow rates, while keeping the air flow rate constant throughout the experiments. The analysis also compared the performance of nanofluids with different concentrations.

### 2.8 Uncertainty analysis

The experimental uncertainty of the cooling performance of the radiator was analyzed considering the uncertainties of the flow rate and temperature differences, following Kline and McClintock procedure, which is commonly used in engineering applications [43].

$$W_{R}={\left[{\left(\frac{\partial R}{\partial_{x1}}w_{1}\right)}^{2}+{\left(\frac{\partial R}{\partial_{x2}}w_{2}\right)}^{2}+\ldots +{\left(\frac{\partial R}{\partial_{xn}}w_{n}\right)}^{2}\right]}^{1/2}$$

The function R, which is dependent on the independent variables x1, x2, …, xn, is associated with the uncertainty of these independent variables, w_1_, w_2_, …, w_n_.

Table 3 summarizes the measurement errors of the main parameters.

**Table 3:** Mesurement parameters and uncertinties.

No	Parameter	instrument	Uncertainties
1	Inlet and outlet temperatures		
	• Air	NTC thermistor	±0.5 °C%
	• Coolant	Resistance thermometer	±0.5 °C
2	Volumetric flow rate of coolant	Turbine flow meter	±3 %
3	Air velocity	Anemometer	±(5%rdg+0.5)

The error analysis of the UA value was found to be 6.1 %, which is considered acceptable according to the literature.

## 3 Results and discussion

After preliminary stability studies revealed that nanoparticles remained in suspension for 8 h, heat transfer experiments were conducted with 5 nanofluids at two different flow rates. In order to evaluate the cooling capability, the radiator's behaviour was monitored throughout testing using thermal imaging, the heat transfer rate was calculated from flow rate and temperature data to evaluate the cooling capability.

### 3.1 Thermal heat analysis

Thermal imaging provides insight into the distribution of temperatures across different sections of the radiator, making it crucial to evaluate the temperature distributions and average temperatures in correlation to enhanced heat transfer. Literature reports imaging results demonstrating that nanofluids, when employed as coolants in radiators, produce more uniform temperature distributions compared to conventional water or ethylene glycol/water mixtures [11,12].

In the experimental study, radiator images were captured with a thermal infrared camera (Fluke TiS60) and thermal heat analysis was performed for each coolant. Temperature distribution was visually represented, with the recording of maximum, minimum and average temperatures.

**Fig. 5:**
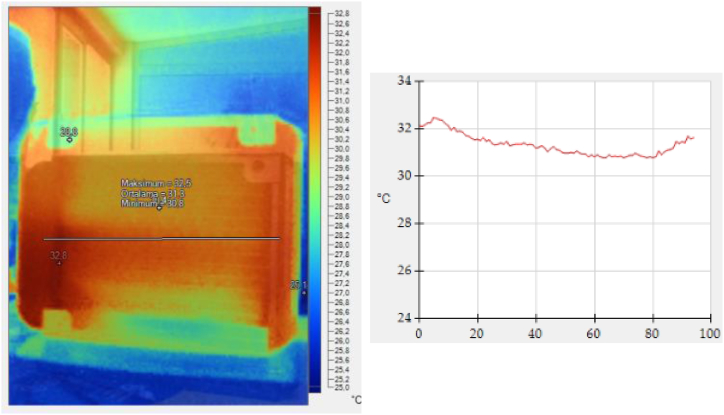
a) Thermal image and b)Temperature distribution of the radiator employing water/EG.

**Fig. 6:**
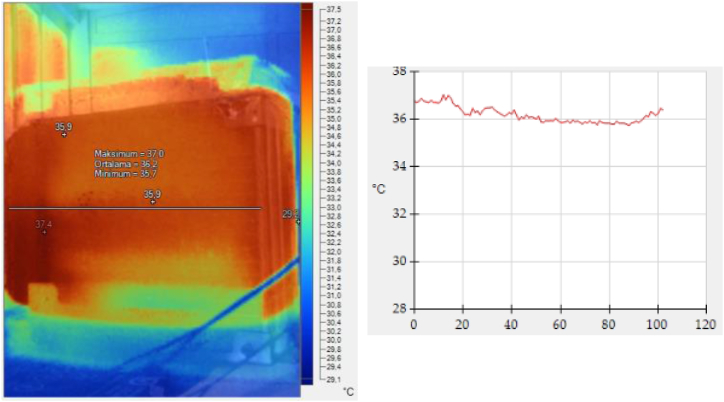
a) Thermal image and b) Temperature distribution of the radiator employing 5.

The thermal images of nanofluids recirculating in the radiator are shown in Fig. 5, Fig. 6. The radiator in Fig. 5 with water/EG has an inlet-outlet temperature difference of 1.7 °C. However, the radiator in Fig. 6 which uses NF5 as the coolant shows a lower temperature difference of 1.3 °C. This marks an improvement of the coolant's thermophysical properties.

Among the prepared coolants, it was observed that NF5 absorbed the most heat. This finding aligns with the outcomes of previous heat transfer experiments, demonstrating an enhancement in the UA value with an increase in the Al_2_O_3_ proportion in the coolant.

The results of the imaging studies have been found to be in alignment with the findings reported in the literature.

### 3.2 Heat transfer analysis

Heat transfer analysis focuses on total heat transfer of a radiator. The efficiency of the heat exchange system is evaluated in this study using the UA value. The overall thermal conductance (UA) values were calculated for the heat exchanger based on the measured inlet and outlet temperatures of the heat exchanger, as well as the mass flow rate of both the coolant and the air.

**Fig. 7:**
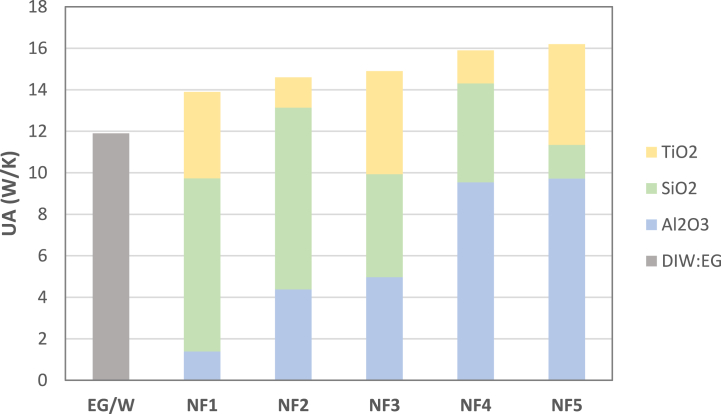
UA values at various concentrations of nanofluids at 0.02 mass flow rate.

UA values for each nanofluid at 0.02 mass flow rate is given at Fig. 7. The UA value for each nanofluid is visualized in proportion to the volumetric ratio of nanoparticles in the nanofluid. Looking at the graph, it can be seen that the largest contribution to the improvement of heat transfer belongs to Al_2_O_3_ and the lowest contribution to SiO_2_. The result also consistent with existing studies in the literature [44,45]. UA values improve with Al_2_O_3_ nanoparticle concentration in coolant. This result is compatible with the specific heat values of the nanoparticles used in the literature [31]. The results obtained are in parallel with Choi et al. in terms of UA in terms of significant improvement with the addition of nanoparticles [46].

**Fig. 8:**
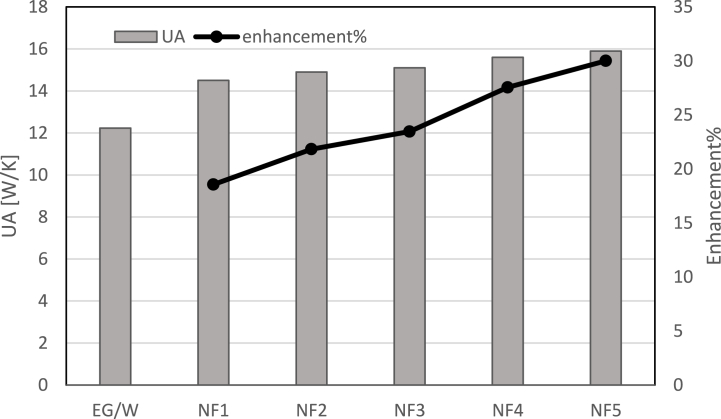
UA values at various concentrations of nanofluids at 0.02 kg/s mass flow rate.

In the second case, the coolant flow rate was increased to 0.02 and the experiments were repeated. The UA values and enhancement ratio of NF at 0.02 mass flow rate is reported in Fig. 8 for NF1, NF2 and NF3 showing the effect of nanoparticles on heat transfer.

The figure reveals that Al_2_O_3_ concentration will cause the UA value and enhancement ratio to increase. At 0.02 kg/s UA value and enhancement ratio compared to EG/W for NF1 is 14.-18.5 %, for NF2 is 14.9–21.8 %, for NF3 15.1–23.4 %, for NF4 15.6–27.5 %, for NF5 15.9–30 %. Gradual enhancement was observed in UA value with increasing Al_2_O_3_ ratio in the coolant. The results obtained in this study are similar to the results of Choi et al. considering that the heat transfer value is significantly improved [46].

**Fig. 9:**
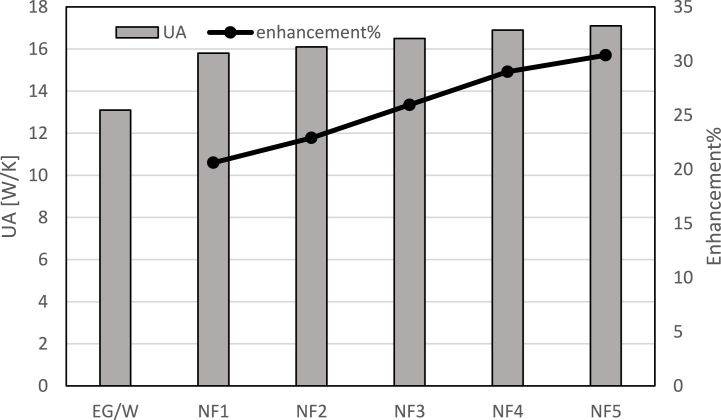
UA values at various concentrations of nanofluids at 0.032 mass flow rate.

Fig. 9 shows the effect of various nanoparticle concentrations on UA and enhancement% at 0032 mass flow rate. UA value increases by 20.6 % for NF1, 22.9 % for NF2, 25.9 % for NF3, 29 % for NF4 and 30.4 for NF5 compared to EG/W.

NF5 configuration has the maximum overall conductance for all mass flow rates.

## 4 Conclusions

As a result of the experiments carried out in the test setup established in the laboratory, it was concluded that the addition of nanoparticles to the coolant increases the heat transfer. The use of nanofluids can lead to an improved cooling performance and a higher energy efficiency of the radiators. In case the stability and sedimentation problems are overcome, nanofluids can be used safely in real application areas and reduce the radiator size.

Our findings demonstrate that the addition of nanoparticles significantly enhances heat transfer performance. Specifically, the enhancement ratio for the nanofluids ranged from 14 % to 30 %, depending on the nanoparticle composition and the flow rate.

• At 0.02 and 0.032 kg/s UA value and enhancement ratio compared to EG/W for NF1 is 14.-18.5 %, for NF2 is 14.9–21.8 %, for NF3 15.1–23.4 %, for NF4 15.6–27.5 %, for NF5 15.9–30 %.
• In the whole range of parameters, the maximum enhancement in the UA value was 30.5 % occurring at 0.032 kg/s mass flow rate using NF5 than the water/EG-cooled system.

The study also highlighted a gradual increase in UA value with increasing Al_2_O_3_ ratio in the coolant. Furthermore, while the water/EG mixture exhibited an inlet-outlet temperature difference of 1.7 °C, the radiator using NF5 demonstrated a reduced temperature difference of 1.3 °C, indicating better thermal performance.

Overall, the use of tri-hybrid nano-coolant has been shown to significantly improve heat transfer and to create a more uniform distribution of temperatures within the radiator, resulting in a lower temperature difference. The observed increase in heat transfer efficiency can be attributed to both the increased flow velocity and the enhanced thermal properties imparted by the nanoparticles. While nanofluids present a promising approach for optimizing radiator systems and potentially reducing radiator size, it is essential to address stability and sedimentation issues for practical applications. Further research is encouraged to explore the long-term stability of these nanofluids and their practical applications in different cooling systems.

## CRediT authorship contribution statement

**Tugba Tetik:** Investigation. **Yasin Karagoz:** Supervision, Investigation.

## Declaration of competing interest

The authors declare that they have no known competing financial interests or personal relationships that could have appeared to influence the work reported in this paper.
